# Functional genomic delineation of TLR-induced transcriptional networks

**DOI:** 10.1186/1471-2164-8-394

**Published:** 2007-10-29

**Authors:** Ran Elkon, Chaim Linhart, Yonit Halperin, Yosef Shiloh, Ron Shamir

**Affiliations:** 1 The David and Inez Myers Laboratory for Genetic Research, Department of Molecular Genetics and Biochemistry, Sackler School of Medicine, Tel Aviv University, Tel Aviv 69978, Israel; 2 School of Computer Science, Tel Aviv University, Tel Aviv 69978, Israel

## Abstract

**Background:**

The innate immune system is the first line of defense mechanisms protecting the host from invading pathogens such as bacteria and viruses. The innate immunity responses are triggered by recognition of prototypical pathogen components by cellular receptors. Prominent among these pathogen sensors are Toll-like receptors (TLRs). We sought global delineation of transcriptional networks induced by TLRs, analyzing four genome-wide expression datasets in mouse and human macrophages stimulated with pathogen-mimetic agents that engage various TLRs.

**Results:**

Combining computational analysis of expression profiles and cis-regulatory promoter sequences, we dissected the TLR-induced transcriptional program into two major components: the first is universally activated by all examined TLRs, and the second is specific to activated TLR3 and TLR4. Our results point to NF-κB and ISRE-binding transcription factors as the key regulators of the universal and the TLR3/4-specific responses, respectively, and identify novel putative positive and negative feedback loops in these transcriptional programs. Analysis of the kinetics of the induced network showed that while NF-κB regulates mainly an early-induced and sustained response, the ISRE element functions primarily in the induction of a delayed wave. We further demonstrate that co-occurrence of the NF-κB and ISRE elements in the same promoter endows its targets with enhanced responsiveness.

**Conclusion:**

Our results enhance system-level understanding of the networks induced by TLRs and demonstrate the power of genomics approaches to delineate intricate transcriptional webs in mammalian systems. Such systems-level knowledge of the TLR network can be useful for designing ways to pharmacologically manipulate the activity of the innate immunity in pathological conditions in which either enhancement or repression of this branch of the immune system is desired.

## Background

Immune systems in vertebrates have two basic arms: innate and adaptive immunity. The innate immune system is the first line of defense protecting the host from invading pathogens such as bacteria and viruses. It consists of various types of leukocytes (e.g., blood monocytes, neutrophils, tissue macrophages, dendritic cells) that specialize in phagocytosis (ingesting and digesting pathogens) and in evoking a complex response at the site of infection, collectively known as inflammation. The adaptive immunity arm is capable of specifically recognizing and selectively eliminating foreign microorganisms and molecules. It relies on T and B lymphocytes that express antigen-specific receptors. Upon encountering their specific antigens, these lymphocytes undergo extensive proliferation (clone expansion), maturation and activation. There are multiple cross-talks between the innate and adaptive immunity arms. For example, the phagocytic cells are intimately involved in the activation of the adaptive arm by functioning as antigen presenting cells (APCs) required for the activation of T lymphocytes, and T_H _lymphocytes secrete stimulatory cytokines that enhance phagocytosis by the specialized phagocytic cells.

Innate immune responses to pathogens are triggered by recognition of prototypical pathogen components, called pathogen-associated molecular patterns (PAMPs), through cellular pattern recognition receptors (PRRs). Prominent among these pathogen sensors is the family of Toll-like receptors (TLRs). To date, ten and thirteen TLR genes have been cloned in human and mouse, respectively; each of the TLRs appears to recognize a unique set of PAMPs [1,2]. TLR1, 2, 4, 5 and 6 are expressed on the cell surface membrane and recognize bacterial and fungal products, while TLR3, 7, 8 and 9 reside in intracellular endosomes and specialize in detection of pathogens' nucleic acids [3]. For example, lipopolysaccharide (LPS), which is a common structure of the cell wall of Gram-negative bacteria, is recognized by the extracellular TLR4, whereas double-stranded RNA (dsRNA), which is a viral PAMP, triggers the intracellular TLR3 signaling. The function of the other TLRs is less characterized.

After recognition of their ligands, TLRs trigger intricate cellular signaling pathways that endow the cells with antiviral and antibacterial states, which are acquired by the induction of protein effectors that impede viral replication and bacteria growth, and of inflammatory cytokines, chemokines and co-stimulatory molecules that enhance the activation of the adaptive immune response [2,4]. The activation of this broad response is mediated by a signaling cascade that leads to stimulation of several transcription factors (TFs), primarily NF-κB, IRF3/7, and AP-1. Important among the induced cytokines are the interferons (IFNs), whose secretion results in the induction of a set of IFN-stimulated genes (ISGs), which are vital components in the development of antiviral and antimicrobial cellular states [5]. The transactivation of the ISGs is controlled via the JAK/STAT signaling pathway either by an IFNα/β-activated TF complex termed ISGF3 (composed of STAT1, STAT2 and IRF9), which binds to a regulatory element denoted as ISRE (IFN-stimulated response element) [5,6], or by an IFNγ-activated STAT1 homodimer complex, which binds primarily to the GAS regulatory element [7].

The transcriptional program spanned by activated TLRs encompasses hundreds of genes. The advent of gene expression microarrays and the availability of complete sequences of the mouse and human genomes enable study of these networks on the system level. Here, we analyzed four publicly available genome-wide datasets that recorded expression profiles in mouse and human macrophages stimulated with various pathogen-mimetic agents, with the goal of obtaining global delineation of the transcriptional network activated by TLRs. Combining computational analyses of gene expression profiles and cis-regulatory promoter sequences, we dissected the TLR-induced transcriptional program into two major components: the first is universally activated by all examined TLRs, and the second is specific to TLR3 and TLR4. Our results identify NF-κB as the key regulator of the universal TLR response and the ISRE element as the key control site of the TLR3/4 specific component, and reveal, on a genomic scale, known and novel target genes regulated by these elements. We also identify novel putative positive and negative feedback loops in these transcriptional programs, further increasing the complexity of the known tightly regulated network induced in response to pathogen invasion. Analysis of the kinetics of the induced network showed that while NF-κB regulates mainly an early-induced and sustained response, the ISRE element functions primarily in the induction of a delayed wave. In addition, we demonstrate that the pair of NF-κB and ISRE elements constitutes a cis-regulatory module that endows its targets with enhanced responsiveness to TLR3/4 activation. By combining expression and promoter analyses, we substantially reduced the high level of noise inherent in genome-wide analysis of such data, and obtained highly reliable results supported by independent datasets from both human and mouse.

## Results

**Figure 1:**
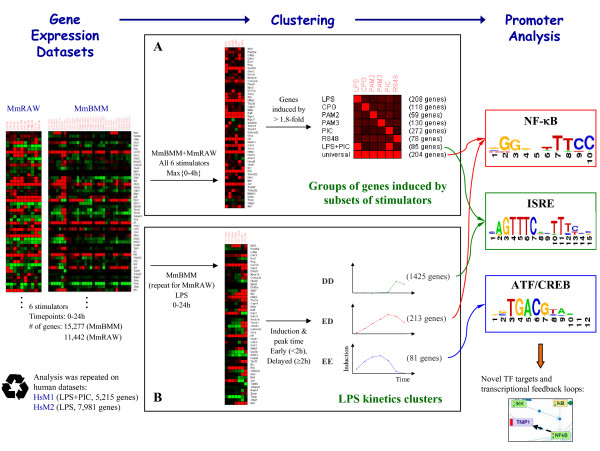
**Analysis Flow**. A schematic sketch of the major steps in our analysis. Using two comprehensive mouse gene expression datasets, we partitioned the genes into distinct groups according to the subset of TLR stimulators to which they were responsive (A), and identified the TFs that control each response group by computational analysis of cis-regulatory promoter elements. We then characterized three kinetic patterns of the transcriptional network induced by LPS treatment (B), and again discovered the TFs that regulate each pattern. A similar analysis of two independent human datasets confirmed our main findings. Integrating the various sources of information points to novel putative targets of the studied TFs, adding new regulatory links to the transcriptional network of the innate immune system.

We sought to obtain a global view of the transcriptional programs that are induced by activated TLRs, and to identify components common to all TLRs and those specific to some of them. To this end, we used four large-scale gene expression datasets that examined global response in mouse and human macrophages stimulated with various TLR stimulators [8-10] (Table 1). Our analysis flow is schematically sketched in Figure 1 and is described in detail in the sections below. In brief, starting with the mouse datasets, we first partitioned the induced genes into disjoint groups according to the subset of stimulators to which the genes were responsive. Applying computational analysis of cis-regulatory promoter elements we sought to discover the major TFs that control each of the identified response groups. Next, we analyzed the kinetics of the transcriptional network induced by LPS treatment, and identified the TFs that regulate each kinetic pattern. Finally, we corroborated the results obtained on the mouse datasets by demonstrating their validity in independent human datasets.

**Table 1:** Summary of datasets analyzed in this study

**Dataset**	**MmBMM**	**MmRAW**	**HsM1**	**HsM2**
**Reference**	Gilchrist et al. (2006) [8]	[11]	Nau et al. (2002) [9]	Jeffrey et al. (2006) [10]
**Organism**	Mouse	Mouse	Human	Human
**Cells**	BMM	RAW264.7	Mph	Mph
**Stimulators**	LPS, CpG, PAM2, PAM3, PIC, R848	LPS, CpG, PAM2, PAM3, PIC, R848	LPS, PIC	LPS
**Time-points**	0 h, 20 m, 40 m, 1 h, 80 m, 2 h, 8 h*, 24 h*	0 h, 1 h*, 2 h*, 4 h, 8 h*, 24 h*	0 h, 1 h, 2 h, 6 h, 12 h, 24 h	0 h, 4 h
**Microarray**	Affymetrix MG430 2.0	Two-channel oligonucleotide chip (Operon)	Affymetrix HU6800	Affymetrix HGU133A
**# distinct annotated genes**	15,277	11,442	5,215	7,981
**Replicates**	Triplicates	Quadrareplicates	One (two at time 0 h)	Duplicates

* time-points measured only for LPS

### Characterization of TLR-induced transcriptional networks

**Figure 2:**
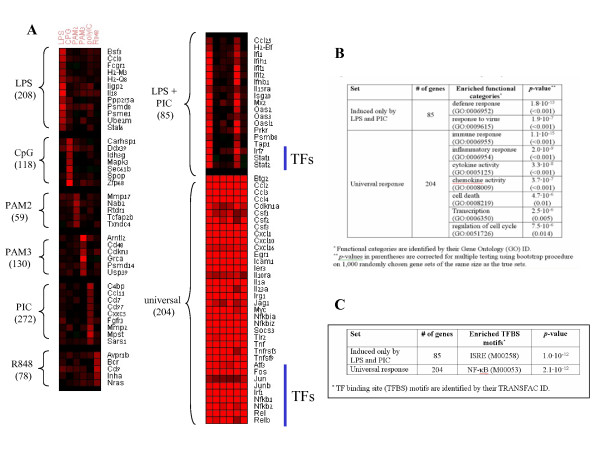
**TLR-induced transcriptional programs**. (A) Genes that were induced by at least one of the six examined TLR stimulators (induction of at least 1.8-fold at any time point) were partitioned into distinct sets according to their agent-induction pattern. Taking into account sets that contained at least 40 genes, only two complex induction patterns were identified in addition to the six agent-specific patterns: universal and LPS-PIC patterns. Selected genes are shown in the heat-map for each set (a complete list of genes is provided in Additional File 1). The maximum induction of the gene over the examined time points per stimulator is depicted in the heat-map. (B) Enriched GO functional categories were identified in the universal and LPS-PIC sets (*p*-values in parentheses are corrected for multiple testing using a bootstrap procedure on 1,000 randomly chosen gene sets of the same size as the true sets). (C) Highly significant over-represented cis-regulatory elements were identified in the promoters of the universal and LPS-PIC sets, pointing to a pivotal role for NF-κB and ISRE in the induction of these two components of the TLR-induced transcriptional program.

In the first step of the analysis, we analyzed the comprehensive gene expression dataset gathered by the Innate-Immunity System-Biology project [11], in which expression profiles were recorded in two murine macrophage cellular systems (bone marrow-derived macrophage cells (BMM) and the RAW264.7 monocyte macrophage-like cell line) at several time points after exposure to six agents, each in a separate experiment. We began with the mouse datasets because they included more stimulators and denser kinetics than the human datasets. The following are the agents examined in mouse, and the TLRs they activate: LPS – TLR4; PAM2 – TLR2:6; PAM3 – TLR1:2; poly I:C (PIC, in short) – TLR3; R848 – TLR7 and TLR8, and CpG – TLR9 (see Table 2). In order to distinguish agent-specific from common responses, we divided the genes into disjoint groups according to the subset of agents in which they were induced. Each group consisted of genes that were up-regulated by at least 1.8-fold (at any time point) by a particular subset of agents, and did not exceed this factor of induction by all other agents (a list of these genes and their group assignment is provided in Additional File 1). In this analysis we included only the time points common to all probed agents: 20 mins, 40 mins, 1 hr, 80 mins and 2 hrs in the MmBMM dataset, and 4 hrs in the MmRAW dataset. Groups with less than 40 genes were ignored, as they do not contain sufficient information for further statistical analysis. Obviously, in such partition some genes are classified somewhat arbitrarily, e.g., a gene whose induction level is slightly above the 1.8 cutoff in LPS and slightly below 1.8 in all other agents, is assigned to the LPS-specific group. However, the mean expression pattern of each gene group reveals a sharp difference between the average induction level in response to the agent(s) that defines the group and the average induction level in response to all other agents (see Additional File 2), indicating that the borderline genes are a minority within the groups. We identified two induction patterns in addition to the six agent-specific sets (Figure 2A): 1) a large core universal response – 204 genes that were induced by all examined stimulators; and 2) a response only to LPS and PIC (which engage TLR4 and TLR3, respectively) – 85 genes that were induced by LPS and PIC, and did not pass the 1.8-fold threshold in the four other stimulators. Remarkably, both of the above sets are substantially larger than all the other non-agent-specific groups (55 groups in total, all of which contained less than 40 genes, with an average size of only 7 genes), pointing to the major biological role of these two response components in the TLR induced network.

**Table 2:** Stimulators used in the mouse MmBMM and MmRAW datasets

**Agent**	**Description**	**Engaged TLR**
**LPS**	Lipopolysaccharide is a component of the bacterial cell wall (gram-negative bacteria)	TLR4
**PAM2**	Synthetic diacylated lipopeptide (mimics bacterial lipoproteins)	TLR2:6
**PAM3**	Synthetic triacylated lipopeptide (mimics bacterial lipoproteins)	TLR1:2
**PIC**	Polyinosine-polycytidylic acid (Poly I:C) is a synthetic mimic of viral double-stranded RNA	TLR3
**R848**	Synthetic molecule of the imidazoquinoline family (mimics a viral product)	TLR7/8
**CpG**	Mimics bacterial and viral CpG DNA motifs	TLR9

Functional characterization utilizing the standard GO ontology [12] revealed that the universal and TLR3/4-specific responder sets were highly enriched for functions related to the innate immune response, including inflammation, and chemokine and cytokine activities (Figure 2B). Interestingly, no enrichment for any functional category was detected for the agent-specific sets. One explanation could be that these sets contain more false positives, as detection of genes induced only in a single condition is more prone to noise. In addition, it is possible that genes specifically induced by a single stimulator are less functionally characterized.

Our next goal was to identify the regulators that underlie the induction of the TLR-mediated transcriptional programs. We and others have demonstrated that combining computational analysis of cis-regulatory promoter elements with gene expression measurements can identify major transcription factors (TFs) that regulate transcriptional networks, even in complex mammalian systems [13-16]. We applied the promoter analysis algorithm PRIMA [14] implemented in the EXPANDER package [17]. Given a target set and a background set of genes, PRIMA performs statistical tests to identify TFs whose binding site (BS) signatures are significantly more prevalent in the promoters of the target set than in the background set. Here, each of the eight gene sets was considered a target set and the entire set of 10,113 genes present on both arrays used in the MmBMM and MmRAW datasets served as the background set (see Methods). PRIMA identified significant over-representation of the NF-κB binding site signature in the group of genes that were induced by all TLRs (p = 2·10^-12^), and of the ISRE element in the set of genes that were induced only by LPS and PIC (p = 10^-12^) (Figure 2C). As in the functional analysis, no over-represented promoter signals were detected for the agent-specific clusters. PRIMA tests are confined to TFs with characterized binding site signatures. Search for novel elements using the MEME motif discovery tool [18] did not find any additional motif, except for the ubiquitous Sp1 signature in several sets. Taken together, the analysis suggests that while NF-κB is universally activated by all TLRs, the TFs that act via the ISRE element (namely, IRF3/7 and the STAT1:STAT2:IRF9 (ISGF3) complex) are activated specifically by the TLR4- and TLR3-mediated signaling pathways. Indeed, many key targets of NF-κB and the ISRE element are in the universal and TLR3/4 sets, respectively, as shown in Figure 2A. Notably, in support of this model, the Nf-κb1, Nf-κb2, Rel and Relb subunits of NF-κB are themselves included in the universal set (that is, they were induced in response to all agents), while Irf7, Stat1 and Stat2, which bind the ISRE, were specifically induced by the LPS and PIC treatments. (Irf9, the third component of the ISGF3 complex, was up-regulated in response to LPS and PIC as well, but only at late time-points – 8 h, 24 h for LPS, 4 h for PIC. As noted above, here we analyzed only time-points 0–4 h, which are common to all the examined TLR-inducing agents.)

Carrying out a similar analysis on the sets of down-regulated genes (using the minimum expression value over time-points 0–4 h in all six agents) did not yield any significant results. However, taking into account the later time-points of 8 h and 24 h (measured only for LPS) identified enrichment of cell-cycle related GO categories and TFs (namely, E2F, NF-Y; data not shown), reflecting proliferation arrest upon pathogen recognition.

### Kinetics of the LPS-induced transcriptional response

**Figure 3:**
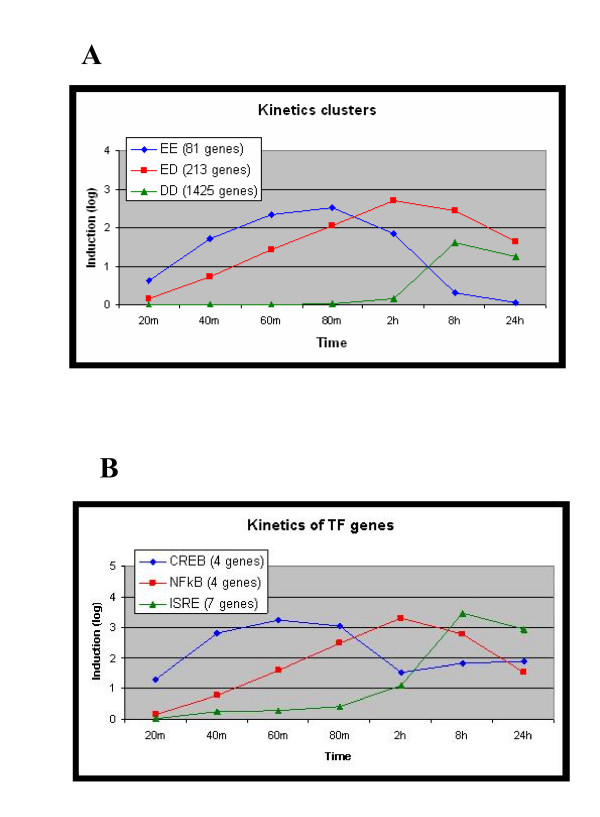
**Kinetics of the LPS-induced transcriptional response**. (A) Genes that were induced by LPS (by at least 1.8-fold) were divided into three kinetic sets according to the time their expression was first induced and the time it peaked. The 'EE' set contains the early induction, early peak genes; the 'ED' set contains early induction, delayed peak genes; and the 'DD' set contains delayed induction, delayed peak genes. The figure displays the mean expression patterns of the genes assigned to the three kinetic sets in the MmBMM dataset (y-axis is log_2 _of induction fold). (B) Mean expression of induced genes that encode for TFs: ATF/CREB (Atf3, Fos, Jun, Junb), NF-κB (Nfkb1, Nfkb2, Rel, Relb), and ISRE (Irf1, Irf2, Irf7, Stat1, Stat2, Stat3, Stat5a). The expression pattern of each TF is highly correlated with that of the kinetic wave, in which the computational promoter analysis found an over-representation of its BSs (compare the kinetic expression of the TF genes (B) and the induced waves (A)).

Expression profiles in response to LPS stimulation were recorded at denser time points (20 mins, 40 mins, 1 hr, 80 mins, 2 hrs, 8 hrs and 24 hrs in the MmBMM dataset, and 1 hr, 2 hrs, 4 hrs, 8 hrs and 24 hrs in the MmRAW dataset), which permitted detailed analysis of the kinetics of the transcriptional program induced by this agent. We partitioned the genes that were induced by LPS (1,719 and 1,239 genes in MmBMM and MmRAW, respectively) into three sets according to the kinetics of their induction, as follows: For each gene we recorded the first time at which it exceeded the 1.8-fold induction threshold, as well as the time at which its expression was highest; we defined three kinetic patterns: 1) Early induction and early peak ('EE' set), containing the genes that peaked (and, obviously, were first induced) before 2 hrs; 2) Early induction and delayed peak ('ED' set) – the genes that were first induced before 2 hrs and peaked at 2 hrs or later; and 3) Delayed induction and delayed peak ('DD' set) – the genes that were first induced (and thus also peaked) at 2 hrs or later (Figure 3A). In both datasets, the 'DD' set was considerably larger than the two other sets, reflecting the fact that the main transcriptional response to LPS exposure was at 2 hrs or later.

Searching for TFs that control these kinetic waves, we applied PRIMA to these six sets (three in each dataset). We identified over-representation of the following BS signatures in both datasets: ATF/CREB in the promoters of genes assigned to the 'EE' set; NF-κB in the 'ED' set; and ISRE in the 'DD' set (Table 3). In addition, enrichment for SRF BS signature was identified in the 'EE' set in MmRAW, and for ETS in the 'DD' set in MmBMM. These results suggest a model in which TFs of the ATF/CREB family modulate an immediate transcriptional response, NF-κB controls an early response that persists longer, and TFs that act via the ISRE element (members of the IRF and STAT families) regulate mainly the delayed transcriptional response. Importantly, in accordance with this model, we observed that genes that encoded for TFs of the respective families followed a kinetic pattern that was correlated with the one manifested by their putative targets (Figure 3 and Table 3). To further corroborate this kinetic model, we carried out a complementary analysis in which we compared the induction kinetics of putative targets of NF-κB and ISRE based on appearance of strong TF binding site (TFBS) motif hits in their promoters (as identified by PRIMA). Comparing the induction of the putative targets of NF-κB or ISRE, but not both (82 and 112 genes, respectively), indeed showed that targets of NF-κB were induced before targets of ISRE (p < 0.01 in both datasets; see Methods). Similar statistical tests showed that genes whose promoter contained an ATF/CREB BS signature peaked at earlier time points than induced genes whose promoter did not contain this cis-regulatory element (p < 0.0001 in both datasets).

**Table 3:** TFBS over-represented in kinetic waves induced by LPS

**Kinetics set**	**Enriched TFBS motifs**	**Dataset**	**# of genes**	*p***-value**
EE	ATF/CREB (M00177)	MmBMM	81	1.0·10^-8^
		MmRAW	100	1.1·10^-5^
	SRF (M00810)	MmRAW	100	4.1·10^-6^
ED	NF-κB (M00053)	MmBMM	213	2.3·10^-7^
		MmRAW	133	2.9·10^-6^
DD	ISRE (M00258)	MmBMM	1425	1.7·10^-17^
		MmRAW	1006	8.4·10^-11^
	ETS (M00971)	MmBMM	1425	1.9·10^-8^

### An additive effect of the pair of NF-κB and ISRE elements

**Figure 4:**
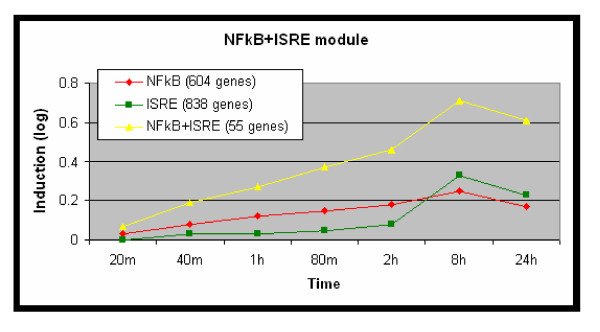
**Identification of the NFkB+ISRE cis-regulatory module**. Mean expression patterns after exposure to LPS (MmBMM dataset) were computed for three disjoint sets of genes – putative targets of each single element separately (604 NF-κB targets, 838 ISRE targets), and targets of both elements (55 genes), obtained by scanning the promoters of all the genes in the MmBMM dataset. Y-axis is average log_2 _of induction fold relative to time 0. Genes whose promoters contain hits for both NF-κB and ISRE elements were more strongly induced by LPS than genes whose promoters contain a hit for only one of these two elements.

The above results suggest that NF-κB and the IRF-like TFs that act via the ISRE element mainly regulate separate components of the TLRs-induced program and different response waves induced by LPS. Yet, genome-wide scan identified 55 genes whose promoters contained hits for these two regulatory elements. In 27 (49%) of these promoters, the ISRE element is located upstream to the NF-κB putative site, indicating no order bias between the two elements. We next examined whether there is an enhanced effect when NF-κB and ISRE elements co-occur; in other words, do genes whose promoter contains both BSs exhibit a unique expression pattern? We did this by comparing the expression of these genes after exposure to LPS to that of putative targets of each single element separately. Targets of the NF-κB+ISRE pair tended to have higher expression values than genes with only one of these elements (Figure 4). Specifically, when the putative targets of NF-κB were sorted in descending order according to their maximal expression value in MmBMM (over all time points), the top 10% genes were significantly enriched for the NF-κB+ISRE pair (p < 0.005; see Methods). The top 10% genes with the ISRE element were also enriched for the pair (p < 0.05). This finding points to an additive effect of these two regulatory elements that boosts the induction of the respective target promoters beyond the induction of genes controlled by only one of them. This suggests that the NF-κB and ISRE cis-elements form together a functional regulatory module in promoters of genes that are induced by LPS. An alternative explanation for this observation is that the identification of targets of a single cis-element is more prone to false-positives than that of both elements, and therefore the expression values we obtained for the set of putative targets of NF-κB and ISRE separately were attenuated to a larger extent by false-positives than the expression of putative targets of the module. However, previous studies support the additive effect of the NF-κB+ISRE module, reporting several genes that were co-regulated by NF-κB and ISRE. Doyle et al [1], for example, experimentally demonstrated functional cooperation between NF-κB and IRF3 in the induction of IFNβ and IP-10 (CXCL10) in response to LPS.

### Corroboration of the findings on independent human macrophage datasets

The results presented hitherto were inferred from analysis of responses of mouse macrophages to various TLR stimuli. Seeking corroboration of our findings in human cells, we analyzed two publicly available datasets that profiled transcriptional responses in immunologically challenged human macrophages. The first study, by Nau et al. [9], examined expression profiles in human monocyte-macrophages at several time points (1 hr, 2 hrs, 6 hrs, 12 hrs and 24 hrs) after stimulation by various agents; among them LPS and PIC are common to the stimuli examined by the mouse datasets we analyzed (this dataset is hereafter called HsM1). The second study, by Jeffery et al. [10] (hereafter called HsM2), profiled transcriptional responses in several human leukocytes challenged with various stimuli, among which monocyte-macrophages treated with LPS for 4 hrs were relevant to our analysis (see Table 1). These two studies provided us with independent data that profiled the transcriptional network induced by activated human macrophages, and allowed us to examine whether our findings on the major roles of NF-κB and ISRE elements in the activation of the transcriptional networks induced by activated TLR4 (LPS) and TLR3 (PIC) are valid also in humans.

Analyzing the HsM1 dataset, we first identified the genes that were induced by LPS alone or by PIC alone, or by both treatments, and subjected these three gene sets to computational promoter analysis. In full accordance with the results obtained on the mouse data, an unbiased search for TFs that underlie the networks induced by LPS and PIC in HsM1 did not identify any signal in the sets of genes that responded specifically to either LPS or to PIC, but did detect a significant over-representation of NF-κB and ISRE elements in the promoters of genes that were induced by both agents (Table 4). This over-representation reflects the superposition of the two components of the TLR-induced transcriptional program: the universal response induced by all TLRs (mediated by NF-κB) and the TLR3/4-specific component (regulated by TFs that act via the ISRE element). These findings were further supported by the second human macrophage dataset that we analyzed: 505 genes were induced by at least 1.8-fold at 4 hrs after LPS treatment in the HsM2 dataset. Unbiased computational promoter analysis again detected only two signals enriched in this gene set: NF-κB (p = 8.8·10^-8^) and ISRE (1.4·10^-12^).

**Table 4:** TFBS over-represented in the response induced by LPS and PIC in the HsM1 dataset

**Set**	**# of genes**	**Enriched TFBS motifs**	***p*-value**
Induced only by LPS	196	---	---
Induced only by PIC	123	---	---
Induced by both LPS and PIC	75	NF-κB (M00053)	1.1·10^-7^
		ISRE (M00258)	8.3·10^-9^

Next, we sought to demonstrate that the kinetic model that emerged in the analysis of the mouse datasets remains valid for the human data. Following the analysis applied to the mouse datasets, we partitioned the genes induced by LPS and PIC in the human datasets to the three kinetic sets: 'EE', 'ED' and 'DD' (again, using the 2 hr time point as the boundary between early and delayed time points), according to the kinetics of their activation, and searched for over-represented signals in the promoters of these gene sets. In agreement with the results obtained on the mouse dataset, here too we observed a strong enrichment for NF-κB and ISRE elements in the 'ED' (early induction, delayed peak) and 'DD' (delayed induction and peak) sets, respectively (Table 5). In contrast to the results found on the mouse dataset (Table 3), we did not detect here an over-representation of ATF/CREB in the 'EE' set (representing early induction and peak). This is probably due to the small size of this set and the existence of only a single "early" time-point (1 hr), which might have hindered statistical detection of enriched signals.

**Table 5:** TFBS over-represented in kinetic waves induced by LPS and PIC in the HsM1 dataset

**Kinetics set**	**TFBS motif**	**Stimulator**	**# of genes**	***p*-value**
EE	---	LPS	12	
		PIC	80	---
ED	NF-κB (M00053)	LPS	34	7.6·10^-7^
		PIC	7	---
DD	ISRE (M00258)	LPS	225	1.8·10^-5^*
		PIC	111	1.9·10^-7^

* a similar TFBS motif of the same element (M00972) received *p*-value 8.4·10^-7^.

Last, we examined whether the additive effect between the NF-κB and ISRE cis-elements could be detected also in the human macrophage datasets. Indeed, the same statistical test we applied to the mouse data revealed that in both HsM1 and HsM2, the 10% most highly induced putative targets of each of the two elements were significantly enriched for genes whose promoter contained a signature for both NF-κB and ISRE (Table 6).

**Table 6:** Statistical significance of increased expression of NF-κB+ISRE module.

**Dataset**	**MmBMM**	**MmRAW**	**HsM1**	**HsM2**
Targets of module vs. targets of NF-κB	0.0045	0.089	0.059	0.0034
Targets of module vs. targets of ISRE	0.041	0.089	0.015	0.0039

The table shows the *p*-values of the enrichment of the module's putative targets within the top 10% targets of NF-κB/ISRE, based on the genes' maximum induction (across all time-points) in response to LPS.

## Discussion

In this study we systematically delineated the transcriptional program induced by stimulation of various TLRs in macrophages. We dissected two major components of this program: the first is a core response universally activated by all examined TLRs, and the second is specifically activated by TLR3 and TLR4. Our analysis identified NF-κB and IRF-like TFs binding ISRE as the key regulators of these two components and pointed to their respective target genes on a genomic scale. While the involvement of NF-κB and IRF-like TFs in response to TLR induction has been known before, our study makes novel contributions to several aspects of system-level understanding of the transcriptional networks induced by innate immunity: (a) the combined, focused reanalysis of four independent datasets identifying a clean, combinatorial response; (b) revealing the intricate kinetics of the transcriptional response; (c) pinpointing novel specific genes involved in each of the responses; (d) identification of NF-κB and ISRE binding site locations over target genes; and (e) the refinement of the understanding of the regulatory circuitry involved in innate immune response.

Novel targets of NF-κB and ISRE identified in this study (see selected examples in Tables 7 and 8) call for experimental validation. Typically, a genome-wide scan for putative TF targets is prone to a high rate of false positives. However, the candidates we identified are based on diverse evidence that collectively increase the confidence that they are true targets: their induction was triggered by several stimulators in multiple time points and in independent studies on two organisms; and in most cases the respective BS signature was identified in both the human and mouse orthologous promoters.

**Table 7:** Predicted NF-κB target genes in the universal TLR response network.

**Symbol**	**NFkB BS (location)**		**LPS maximum induction (log_2_)**				**LPS**	**Validated**
	**Human**	**Mouse**	**MmBMM**	**MmRAW**	**HsM1**	**HsM2**	**kinetics**	**BS**
**CXCL10**	GGGAAATTCC (-176)	GGGAAATTCC (-233)	10.42	5.47	7.98	6.9	ED	[41]
**RELB**	GGGGTTTTCC (-107)	GGGGTTTTCC (-96)	4.3	1.27	0.77	1.46	ED/EE*	[42]
**NFKBIA**	TGGAAATTCC (-84)	GGGAAACCCC (-81)	4.1	4.25	3.35	2.58	ED	[43]
**NFKB2**	GGGAATTCCC (-101,-73)	CGGGAATTCC (-102,-74)	3.56	3.82	1.17	1.72	ED	[44]
**SDC4**	N/F	GGGGAATTCC (-81)	1.53	2.78	1.01	2.02	DD/ED*	[45]
**CD69**	GGGAAAATCC (-222)	GGGAAAATCC (-220,-155)	8.3	1.82	0.88	3.11	ED/DD*	[46]
**BIRC3**	GGAAATCCCC (-177)	GGAAATCCCC (-60)	2.97	0.75	3.19	4.03	ED	[47]
**MAP3K8**	GGAAAACCCC (-724)	CGGAATTTCC (-490)	3.42	0.46	0.65	2.98	ED	---
**BATF**	N/F	GGGATTTTCC (-233)	4.51	3.07	3.31	1.04	DD	---
**IRG1**	N/F	TGGAAATTCC (-50)	10.8	7.69	x	x	ED	---
**RIPK2**	GGGGCTTTCC (-310)	GGGATTTTCC (-521)	2.51	x	x	3.23	ED	---
**GCH1**	CGGGCTTTCC (-11)	N/F	3.19	0.82	6.19	3.64	ED/DD**	---
**TNIP1**	GGGGACTTTC (-68)	N/F	3.51	-0.64	3.01	2.46	ED/DD**	---

Promoter sequences matching the PWMs of NF-κB and ISRE were identified by the PRIMA software; mapping between human and mouse orthologous genes was downloaded from the Ensembl web-site; sequences are shown on the coding strand; in cases of multiple matches, the sequence of the first listed match is shown; "N/F" means no putative BS was found. For each gene, the tables indicate (the log_2 _of) its maximum fold-induction (over all time-points) in response to LPS in all four datasets, as well as its kinetic pattern (in case of conflicting patterns in different datasets, the default pattern is in MmBMM, * is in MmRAW, and ** is in HsM1). References for validated BSs are given in the last column.

**Table 8:** Predicted ISRE target genes in the specific response to LPS and PIC

**Symbol**	**ISRE BS (location)**		**LPS maximum induction (log**_2_**)**				**LPS kinetics**	Validated BS
			
	**Human**	**Mouse**	**MmBMM**	**MmRAW**	**HsM1**	**HsM2**		
**IFNB1**	GGGAGAAGTGAAAGT (-59)	GGGAGAACTGAAAGT (-150)	5.53	0.44	3.28	x	ED/DD**	[2]
**TOR3A**	GCGGTTTCATTTCCC (161)	ACTGTTTCATTTTCC (-485)	4.08	2.31	x	-0.19	DD	[48]
**OAS3**	GAAAGAAACGAAACT (-29,108)	GGAGAAAACGAAAGT (-77,0)	5.21	2.85	x	2.42	DD	[49, 50]
**OAS2**	TCAGTTTCAGTTTCC (49)	TGAGTTTCGATTTCC (-74)	3.24	2.1	2.56	2.53	DD	[50]
**OASL**	TTGAGAATCGAAACT (-288)	CACAAAAGAGAAACT (-159)	7.93	5.79	2.7	3.98	ED/DD**	[50]
**CFB (BF)**	CTTGTTTCACTTTCA (-98)	ATAGTTTCTGTTTCC (-148)	8.38	3.32	2.04	x	DD	[51]
**TRIM21**	GCGGAAACTGAAAGT (9)	GAGGAAACTGAAAGT (-30,4)	2.65	1.52	2.73	0.22	DD	[52]
**IFIH1**	ATCGAAACAGAAACC (-178)	ATCGAAACAGAAACC (-65)	4.55	2.92	x	3.09	DD/ED*	---
**NMI**	N/F	ACCGAAAGTGAAAGT (71)	3.31	1.54	1.61	1.36	DD	---
**LGP2**	TCAGTTTCAGTTTCC (-1)	TCAGTTTCATTTCTA (-1)	1.87	2.07	x	x	DD	---
**RTP4 (IFRG28)**	ACAGAAACAGAAACT (-39,-15)	TTGGAAACCGAAACT (-84,-58,-35)	2.65	1.59	x	2.33	DD	---
**BATF2**	GGAGAAACTGAAACT (-2)	GGAGAAACTGAAACT (-95)	5.64	1.9	x	x	DD	---
**STAT2**	CTAGTTTCGGTTCCG (-353)	CTGGTTTCAGTTTCC (-303)	5.94	2.01	1.5	1.5	DD	---

See legend of Table 7.

The repertoire of the TLR universal response includes pro-inflammatory cytokines and chemokines (e.g., Ccl2-4, Csf1-3 and Cxcl1, which orchestrate innate immunity fight against pathogens), as well as co-stimulatory molecules (e.g., Il23a) that promote the activation of the T-cell branch of the adaptive immunity. The universal response also contains many general stress-responsive genes (e.g., Jun, Fos, Atf3, Egr1-3, Myc) that control cell proliferation and survival. Prominent among the genes specifically induced by TLR3 and TLR4 are the interferon (IFN)-induced genes (Figure 2A). IFN-induced genes comprise potent antiviral molecules (e.g., Mx2, Isg20, Oas2-3, Prkr) and are therefore expected to be induced by TLR3, which is activated by virally derived dsRNA. However, IFNs also have an important role in linking innate and adaptive immunity by regulating the induction of genes that enhance T-cell activation and antigen-presentation capacity in response to pathogen infection (e.g., Il15, Tap1, Psmb8), which explains their induction by bacterial stimuli such as LPS [19,20].

**Figure 5:**
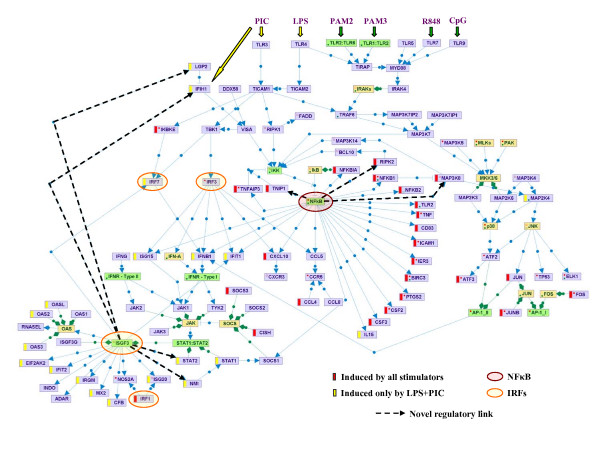
**TLR-induced signaling pathways and transcriptional programs**. The map, constructed using our SPIKE knowledge-base of signaling pathways [40], presents current knowledge on signaling cascades emanating from activated TLRs and culminating in activation of several key TFs and their respective target genes to achieve robust antiviral and antimicrobial responses. SPIKE maps contain nodes representing three biological entities: gene/proteins (violet nodes); protein complexes (green nodes, e.g., the ISGF3 complex); and gene families (yellow nodes, e.g., the IκB family of NF-κB inhibitors). The map contains two types of edges: Blue edges represent regulations between genes/proteins. Arrowheads (→) correspond to activation, and T-shaped edges (---|) represent inhibition. Green edges represent containment relations between nodes (e.g., the relationships between a complex and its components). Red and green dots within a node indicate that not all the regulation and containment relations stored in SPIKE's DB for that node are displayed on the map. Genes that were universally induced by all examined TLRs are marked by a red bar to the left of the node; genes that were specifically induced by LPS and PIC (which activate TLR4 and TLR3, respectively) are marked by a yellow bar. Novel regulatory links identified in this study that close feedback loops within the TLR-induced network are emphasized in the map by a dashed arrow.

Without any prior knowledge on TLR signaling, our computational promoter analysis revealed NF-κB as the pivotal regulator of the universal-TLR transcriptional response. This finding is in line with current biological knowledge. Several molecular mechanisms through which NF-κB is activated by TLR signaling have been characterized [2,20]. The first depends on Myd88 and is utilized by all TLRs with the exception of TLR3. Activated TLRs recruit Myd88, which then associates with members of the IRAK family, initiating a cascade in which TRAF6 and TAK1 (official symbol: MAP3K7) are sequentially activated. TAK1 in turn promotes downstream activation of the IKK complex, which leads to the activation of NF-κB by directly phosphorylating, and thereby removing the inhibitory effect of, the members of the IκB family on NF-κB (Figure 5). On the other hand, TLR3 activates NF-κB in a Myd88-independent manner: The TRIF adaptor protein (TICAM1) is recruited to activated TLR3, and then directly interacts with TRAF6, which presumably leads to the activation of NF-κB using the same cascade described above for the Myd88-depndent pathway [20] (Figure 5). Substantiating the universal role of NF-κB in the TLR-induced network, we observed that the NFκB1, NFκB2, Rel and Relb subunits of the NF-κB heterodimer were induced by all examined stimuli.

Superimposed on the TLR universal program, we detected a robust TLR3/4-specific response, and demonstrated by promoter analysis that its key regulator is the ISRE element. In addition, our results indicate that this ISRE-mediated response is kinetically delayed compared to the NF-κB-regulated program. These findings too are corroborated by current biological knowledge. The ISRE cis-element is bound by members of the IRF and STAT TF families. Several studies demonstrated the existence of two waves of activation of TFs that act via ISRE by TLR3 and TLR4 [1,20-22]. The emerging model is that IRF3, which is post-translationally activated by TLR3 and TLR4 via a cascade that involves the TRIF (TICAM1) and TRAM (TICAM2) adaptor proteins and their downstream kinases IKKε (IKBKE) and TBK1, promotes an early wave of IFN-β gene induction (Figure 5) [1,23]. Once IFN-β is produced and secreted, it engages the type-I IFN receptor in both paracrine and autocrine fashion, thereby triggering the JAK-STAT signaling cascade that culminates in the activation of the ISGF3 TF complex, which is comprised of STAT1, STAT2 and IRF9 (official symbol: ISGF3G) [24]. ISGF3 induces the expression of IRF7, which in turn further activates the expression of type-I IFNs. In this way, a positive loop is established, which ensures persistent expression of IFN-stimulated genes that enhance the antiviral and antimicrobial cellular state [20]. Strikingly, in full compliance with this model, we observed that IFN-β and the IRF7, STAT1 and STAT2 TFs were specifically induced by LPS and PIC in the datasets we analyzed (Figure 5).

Our analysis points to novel feedback loops in the TLR-induced network, further increasing the known complexity of the regulatory circuits that modulate its induction and repression (see Figure 5 and Tables 7, 8): We identified IFIH1 (also known as MDA5) and LGP2 as novel putative targets regulated by the ISRE element. IFIH1 is a non-TLR cytoplasmic sensor that detects actively replicating viruses [2,25], and triggers the induction of the NF-κB and IRF3 pathways via the activation of the adaptor protein VISA (also known as cardif or IPS-1) [26]. Moreover, it has been recently demonstrated that IFIH1 detects cytoplasmic dsRNA generated during viral replication (while TLR3 detects viral dsRNA phagocytosed in endosomes), and that this sensor also binds to PIC and mediates type I IFN responses to this synthetic analog of viral dsRNA [27]. Therefore, the transcriptional program induced by PIC stimulation probably reflects a combined outcome of the activation of TLR3-mediated and IFIH1-mediated pathways.

Interestingly, the second putative ISRE target we identified, LGP2, is a direct negative regulator of IFIH1 [28]. The simultaneous activation of positive and negative regulators of the same pathway seems to be a recurrent theme in the logic of cellular signaling networks. Another novel putative positive loop in the ISRE-regulated network is mediated by NMI, which enhances the transcriptional activity of STAT-1 [29]. In the NF-κB-regulated transcriptional response, which is universally activated by all examined TLRs, we identified MAP3K8 (also known as TPL-2 and COT) and RIPK2 as novel targets that form positive feedback loops which reinforce the persistent activation of this network [30,31], and TNIP1 as a regulator that forms a negative feedback loop which inhibits the IκK complex, thereby contributing to the turning-off of this response [32].

The kinetic analysis of the response to LPS also suggests a role for the ATF/CREB cis-regulatory element. We identified a significant over-representation of this signature on promoters of genes whose expression peaked at very early time points (before 2 hrs). Two alternative interpretations of the role played by these elements are consistent with this rapid pattern of induction: According to the first, members of the ATF/CREB family activate this early and very short response; the second interpretation ascribes an inhibitory effect to these elements, implying that the TF(s) that act via them repress the expression of their target genes, and therefore the induction of these targets declines shortly after their activation. A recent study by Gilchrist et al. [8] demonstrating that ATF3 negatively regulates a subset of NF-κB target genes induced by TLR4 supports the second interpretation. Notably, the ATF3 gene itself is included in the TLR universal response, pointing to a negative loop that regulates a sub-network of TLR-induced transcriptional program.

The computational promoter analysis ferreted out the major regulators of the two components of the TLR-induced network. This complex transcriptional network is likely regulated by additional TFs, which were not detected by promoter analysis. Indeed, the TLR universal response contains several other TFs in addition to those discussed above (e.g., Egr1-3, c-Myc, Ets2, Fos). This could be explained by the fact that our statistical promoter analysis detects TFs with a relatively high number of direct targets, whose BSs are located within the scanned promoter region and which were responsive beyond a certain threshold in the studied conditions. It is therefore expected to miss TFs that: (a) have a small number of directly induced targets; (b) bind at large distances from the transcription start site; (c) regulate the TLR network by interacting with other TFs rather than directly binding to the DNA; or (d) have a very subtle (though, perhaps, biologically important) influence on the expression of their targets.

Our results suggest mainly distinct programs mediated by the NF-κB and ISRE cis-elements. However, when the two elements co-occured in the same target promoter, we detected an additive effect that boosts the induction of the target genes. This finding further defines the NF-κB+ISRE pair as a functional transcriptional module, and adds several novel candidates to the list of genes reported to be controlled by it [1,33-35] (Tables 7, 8). Importantly, IFN-β is among the genes whose promoters were empirically demonstrated to be under the regulation of the NF-κB+ISRE pair [1].

## Conclusion

Our analysis demonstrates the power of functional genomics approaches to delineate intricate transcriptional networks in mammalian systems. Microarray data are often noisy and do not distinguish between direct and secondary responses. Likewise, large-scale promoter scanning for putative TF targets produces many false positives due to the short and degenerate nature of BS signatures. Combining these two sources of information, and augmenting them by utilizing datasets and promoter sequences from both human and mouse, gave us an accurate, system-level delineation of the TLR-induced transcriptional program, and identified highly reliable putative direct targets of its key regulators. The findings reported in this study generalize, on a genomic scale, the current knowledge on the identity, function, kinetics and modular organization of the transcriptional regulators that mobilize the innate immune response, which is often based on studies of specific genes. Such knowledge can be useful for designing ways to pharmacologically manipulate the activity of the innate immunity in pathological conditions in which either enhancement or repression of this branch of the immune system is desired.

## Methods

### Microarray datasets

The four expression datasets analyzed in this study are summarized in Table 1. We used the original normalized probe expression values, as provided by the authors. In each dataset, we averaged measurements over replicate samples, and then, for each probe, we divided expression values in treated samples by the values in the corresponding control samples (time 0 hr). These fold-change ratios were log (base2)-transformed and averaged over probes that correspond to the same gene. Mapping probes in the MmBMM, HsM1 and HsM2 datasets to Ensembl gene ids was done using annotation files provided by Affymetrix. The MmRAW dataset included the Entrez-Gene id of each probe; we used Biomart [36] to map Entrez-Gene ids to Ensembl gene ids. The HsM1 experiment measured responses of macrophages cultured with LPS derived from *E. coli *(LPS_E) and *Salmonella typhi *(LPS_S). We regarded LPS_E and LPS_S as duplicates and averaged over these two conditions.

### Definition of stimulator-induced genes

In all datasets except HsM1, a gene was considered to be induced by a given stimulator if its expression level in one or more of the time points was at least 1.8-fold higher than its expression at time 0. The results we report are not sensitive to the chosen cutoff and remained consistent for a wide range of values (from 1.5- to 2-fold). In HsM1 we used a more stringent threshold of 3.5-fold, since the expression values in this dataset showed a much larger variance, probably because no replicates were performed (except for time 0). This threshold was chosen so that a similar percentage of the genes will be considered induced in HsM1 as in the other datasets.

### Groups of genes induced by subsets of stimulators

The two mouse datasets – MmBMM and MmRAW – share 10,113 genes. Using the maximum induction-fold of each of these genes, computed over six time-points (20 mins-2 hrs in MmBMM, and 4 hrs in MmRAW), for each of the six stimulators (LPS, PAM2, PAM3, PIC, R848 and CpG), we partitioned the genes into groups as follows. We enumerated all 63 (= 2^6^-1) non-empty subsets of the six stimulators, and for each such subset we collected all the genes that were induced in those stimulators and not induced in the others. Ignoring sets with less than 40 genes, we obtained eight gene sets (Figure 2A): six agent-specific sets (i.e., genes that were induced only in one of the six stimulators), an LPS-PIC specific set, and a universal response set.

In humans, we repeated the above analysis for the LPS and PIC stimulators in the HsM1 dataset. Here, we used all five time-points (1 hr–24 hrs), and an induction threshold of 3.5-fold (see Table 4).

### Functional categories analysis

Identification of enriched Gene Ontology (GO) biological processes categories was done using the TANGO algorithm implemented in the EXPANDER package [17]. In brief, TANGO calculates the statistical significance of GO categories' over-representation within a given set of genes by computing the upper tail of the hypergeometric distribution. In order to account for multiple testing, a major challenge in such an analysis due to the strong dependencies among GO categories, TANGO estimates fixed *p*-values using an empirical distribution based on 1,000 randomly chosen gene sets. We report all GO categories with an enrichment *p*-value less than 10^-5 ^(before correcting for multiple testing) (see Figure 2B). Association of mouse genes with GO categories was downloaded from the GO web-site [37] (Sep 2006).

### Computational promoter analysis

Identification of enriched BS signature of known TFs was done using our PRIMA algorithm [14], which is implemented in the EXPANDER package. PRIMA identifies TFs whose BS signatures are significantly abundant in the promoters of a specified group of genes, given their distribution in the promoters of the entire background set (i.e., all the genes present on the chip). PRIMA uses position weight matrices (PWMs) as models for regulatory sites that are bound by TFs. 498 PWMs that represent human or mouse TFBSs were obtained from the TRANSFAC database (release 10.2, June 2006) [38]. Promoter sequences corresponding to all known human and mouse genes were extracted from the Ensembl project (release 40, Sep 2006) [39]. PRIMA scanned both strands of each promoter sequence in the region from 600 bps upstream to 100 bps downstream of the putative transcription start site (TSS). Repetitive elements were masked out. A detailed description of how PRIMA determines PWM cutoffs, identifies putative TFBSs, and computes enrichment scores is given in [14]. We report TFs with an enrichment *p*-value less than 10^-5^. We used this stringent threshold due to the large number of PWMs examined. Note, however, that there is a very high level of redundancy in the TRANSFAC database. For example, there are seven different PWMs for NF-κB, which are naturally all very similar. Thus, the actual number of independent multiple tests performed by PRIMA is considerably less than the total number of PWMs. For each of the TFs reported in this study, we chose the PWM that gave the best overall results (in terms of enrichment): M00053 for NF-κB, M00258 for ISRE, and M00177 for ATF/CREB; other PWMs of these TFs often gave very similar *p*-values.

We also subjected each of the eight TLR-induced gene sets (Figure 2) to the MEME program (version 3.0.3) [18]. MEME is a tool for discovering motifs de-novo in a group of related DNA sequences. MEME was run with a 4^th^-order Markov background model, which we constructed using all the mouse promoter sequences (from 600 bps upstream to 100 bps downstream the TSS). We searched for motifs of length 8 and 10, and used the following options: "-dna -revcomp -mod zoops -evt 0.001 -text -nostatus".

### Statistical tests for the kinetics of TF targets

In order to statistically evaluate the difference in the induction time of NF-κB and ISRE targets, we counted the number of putative targets of these elements, denoted *s*_1 _and *s*_2_, respectively, that were induced up to 1 hr after LPS treatment. (genes whose promoter contained both the NF-κB and ISRE signatures were ignored in this test). Given the total number of putative targets (induced at any time-point), denoted *t*_1 _and *t*_2_, respectively, we computed the probability that out of *s*_1 _+ *s*_2 _early-induced genes, at least *s*_1 _of them are targets of NF-κB. A small probability indicates that statistically significant number of the early-induced genes is regulated by NF-κB. This probability is given by the hypergeometric tail distribution:

(1) $$P=\sum_{i=s_{1}}^{\min\left\{t_{1},s_{1}+s_{2}\right\}}\frac{\left(\begin{array}{c} t_{1}\\ i \end{array}\right)\left(\begin{array}{c} t_{2}\\ s_{1}+s_{2}-i \end{array}\right)}{\left(\begin{array}{c} t_{1}+t_{2}\\ s_{1}+s_{2} \end{array}\right)}$$

Using a similar statistical test, we showed that the peak time of putative targets of ATF/CREB is significantly earlier than that of all other induced genes. Denoting by *t*_1 _(*t*_2_) the number of LPS-induced genes that are (are not) putative targets of ATF/CREB, out of which *s*_1 _(*s*_2_) reached their maximal expression at or before 1 hr, we computed the hypergeometric probability as above.

### Statistical evaluation of increased induction of targets of NF-κB+ISRE

To examine whether there is a significant additive effect between the NF-κB and ISRE elements, we performed the following test: Given the total number of genes whose promoter contains signatures of both NF-κB and ISRE, or only NF-κB, denoted *t*_1 _and *t*_2_, respectively, we checked whether there is an enrichment of NF-κB+ISRE joint targets within the 10% most highly induced NF-κB targets. Here, genes were ranked based on their maximum induction in response to LPS. Let *s*_1 _and *s*_2 _denote the number of NF-κB+ISRE and NF-κB (but not ISRE) targets, respectively, whose induction-fold is above the aforementioned 10% threshold (i.e., *s*_1 _+ *s*_2 _= (*t*_1 _+ *t*_2_)/10). Then, using the standard hypergeometric score (Equation 1), we computed the probability to observe at least *s*_1 _highly-induced NF-κB+ISRE targets, given *t*_1_, *t*_2 _and *s*_2_. For example, in the MmBMM dataset, we found an NF-κB signature in 659 genes, of which 55 also contained an ISRE element; among the 65 NF-κB targets with highest induction by LPS, 12 genes also had an ISRE element.

Thus, *t*_1 _= 55, *t*_2 _= 604, *s*_1 _= 12, and *s*_2 _= 53, which gives *p *= 0.004.

The above test evaluates the increased expression of putative targets of the pair NF-κB+ISRE with respect to all NF-κB targets. We performed a similar test to check the increased expression of NF-κB+ISRE relative to all ISRE targets.

## Authors' contributions

RE and CL conceived the study, carried out the analyses and drafted the manuscript. YH participated in the data analysis and developed the methods for the kinetic analysis. RS and YS participated in the design of the study, led and funded it and reviewed the manuscript. All authors read and approved the final manuscript.

## Supplementary Material

Additional file 1 The file lists the genes that were induced by TLR activation and their assignment into the agent specific, LPS+PIC and universal clusters. Click here for file

Additional file 2 The mean expression pattern of each gene cluster (representative genes of each cluster are shown in Figure 2A). Click here for file
